# Patient satisfaction with nurses' care is positively related to the nurse–patient relationship in Chinese hospitals: A multicentre study

**DOI:** 10.3389/fpubh.2022.1109313

**Published:** 2023-01-16

**Authors:** Shujie Guo, Yulan Chang, Hongwei Chang, Xiaoxiao He, Qiuxue Zhang, Baoyun Song, Yilan Liu

**Affiliations:** ^1^Henan Provincial People's Hospital, Zhengzhou, Henan, China; ^2^Department of Nursing, Henan Vocational College of Nursing, Anyang, Henan, China; ^3^Nursing College of Tongji Medical College, Huazhong University of Science and Technology, Wuhan, Hubei, China; ^4^Department of Anesthesiology, Qilu Hospital of Shandong University, Dezhou Hospital, Dezhou, Shandong, China; ^5^Department of Nursing, Henan Province People's Hospital, Zhengzhou, Henan, China; ^6^Department of Nursing, Union Hospital of Tongji Medical College, Huazhong University of Science and Technology, Wuhan, Hubei, China

**Keywords:** Chinese, hospital patients, patient satisfaction, nurse–patient care, nurse–patient relationship

## Abstract

**Background:**

The nurse–patient relationship and nursing care satisfaction are important factors that represent whether patients experience the care they expect from nurses. However, research is lacking on the relationship between nursing staff and patients, and the correlation between nursing care satisfaction and relationship care in China. Therefore, this study aimed to explore the correlation between the nurse–patient relationship and patients' satisfaction with nursing care, to form a basis for corresponding intervention measures.

**Methods:**

A total of 29,108 patients from 107 hospitals in 30 provinces/municipalities in China completed a general information questionnaire, the Nursing Care Satisfaction Scale, and Relational Care Scale.

**Results:**

The average nurse–patient relational care scale score was 4.38 ± 0.57, and the average patients' satisfaction with nursing care scale score was 5.40 ± 0.86. Nursing care satisfaction score was significantly related to differences among patients in different age, gender, marital status, education level, occupation, residence, family per capita monthly income, type of medical insurance, medical department, and regional patient characteristics. The correlation analysis showed that the total nurse–patient relational care score and its three dimensions of caring, trust, and professional ethics correlated positively with nursing care satisfaction scores. The multiple linear regression analysis showed that patients' age, marital status, region, department, income, type of medical insurance and the caring, trust, and professional ethics dimensions of relational care predicted nursing care satisfaction.

**Conclusion:**

Enhancing nurse–patient relational care improves nursing care satisfaction, reduces nurse–patient disputes, promotes early rehabilitation of patients, and ensures patient safety.

## 1. Introduction

Humanistic care is the core and essence of nursing, which includes the care provided by nurses to patients, nursing managers to nurses, mutual care between nurses, and the self-care of nurses (1–4). Advocating humanistic care in nursing helps build a harmonious nurse–patient relationship, improve the quality of nursing services and patients' satisfaction, and enhance nurses' professional recognition (5–8). The nurse–patient relationship and nursing care satisfaction are important factors that refer to whether patients experience the care they expect from nurses (9, 10). The content of caring research is very broad, as foreign and domestic research mainly involves the following aspects: the nature of care, care in clinical nursing, care behavior evaluation of nursing staff and patients, care ability evaluation, care efficiency evaluation, organizational care atmosphere evaluation, and patients' experience of care (11, 12). However, there is a lack of research on the relationship between nursing staff and patients, and the correlation between nursing care satisfaction and relational care. An extensive literature search revealed a scarcity of research on the above topics at present and most were single-center studies with small sample sizes (13–15). Acknowledging the above, numerous humanistic nursing training programs have gradually been carried out in China. What is more noteworthy is that the Humanistic Care Professional Committee of Chinese Association for Life Care issued the Humanistic Care Management Specification for Ward Nursing in April 2022, which further provides concrete and feasible guidelines for humanistic care services in China's hospitals (16). Based on the above research background, we conducted a cross-sectional survey *via* the Humanistic Care Professional Committee of Chinese Association for Life Care to understand the current situation and risk factors relating to humanistic care in Chinese hospitals at all levels, so as to provide a basis for the solid practice of humanistic care in medical institutions. This study also analyzed the correlation between the relationship between the nurse–patient relationship and patients' nursing caring satisfaction, so as to provide a basis for the establishment of a relationship care system between nurses and patients, and achieve the aim of improving nursing care satisfaction among patients.

## 2. Methods

### 2.1. Survey participants

Ethical approval for this study was obtained through our university's Research Ethics Board (2022-S161). The data for this study were obtained from the database established by the expert consensus of the “Norms of Practice of Humanistic Care in Hospital Nursing” and the group standard application unit of the “Norms of Humanistic Care Management in Ward Nursing” of the Humanistic Care Professional Committee of Chinese Association for Life Care. Considering the regional distribution, multi-stage stratified sampling was used to recruit participants from July 1 to August 15, 2022. In the first stage, four regions, namely northeast, east, central, and west, were selected, as well as provinces or autonomous regions and municipalities. In the second stage, hospitals of Grade II and above in each province were taken as units (according to the survey, the managers of the surveyed hospitals were members of the National Humanistic Nursing Special Committee). In the third stage, the managers of the hospitals who were selected during the second stage were selected as units, and 107 hospitals nationwide were included. The following patient inclusion criteria were used: (1) All participants were outpatients or stable inpatients; (2) All participants were aged above 18 years old or their legal guardians were older than 18 years old; (3) Study participants or their legal guardians provided informed written consent. The following exclusion criteria were used: (1) Patients unable to complete the cognitive assessments required for the trial; (2) Patients without smartphones or who cannot answer the questionnaire using a smartphone. Informed consent was obtained from all respondents. We determined that the sample size should be at least 5–10 times the questionnaire's total number of 35 items. A sample of 350 was determined as the total needed, and a 10% loss rate was taken into account. Therefore, we planned to recruit at least 385 patients in each hospital. The actual number of patients who were investigated in this study was 31,095 from 107 hospitals in China, of which 29,108 valid questionnaires were returned.

### 2.2. Survey tools

#### 2.2.1. General information questionnaire

The researchers developed the design and included the patients' hospital, gender, age, marriage, education level, place of residence, medical insurance type, family monthly income, department visited, region, and whether surgery was performed.

#### 2.2.2. Nursing care satisfaction scale

The scale designed by the Nursing Care Quality Control Committee of Houston Health Care System (17) was used. This scale consists of 20 items and covers 12 aspects, including nursing coordination, nursing ability, teaching/learning ability, emotional support, respect for individuality, physical comfort, availability, helping/trusting relationship, patient/family involvement, physical environment, spiritual environment, and outcome. The patients' satisfaction with the humanistic care of nurses was investigated, and the patients were asked to evaluate the care they received during their last hospitalization. For each item, “never,” “rarely,” “sometimes,” “often,” “most of the time,” and “always” were scored from 1 to 6 points, giving a total score of 120. The higher the score, the higher the patient's satisfaction with the nurses' care. The Cronbach's α coefficient of this scale in this study was 0.98, showing good reliability.

#### 2.2.3. Relational Care Scale

This scale was originally developed by Ray and Turkel in 2001 according to the grounded theory and then verified and improved through a series of qualitative and quantitative studies that demonstrated good reliability and validity in foreign countries (18). The patient version consists of 15 items that could be divided into the following three dimensions: work ethics, trust, and care. Each item was scored from 1 to 5 points from “strongly disagree” to “strongly agree.” The total score is 75 points. The higher the score, the better the nurse–patient relationship. This study was the first to use the scale in China, and the reliability of the scale was good. The Cronbach's α coefficient of the overall scale was 0.98, and the Cronbach's α coefficients of trust, care, and professional ethics were 0.97, 0.98, and 0.95, respectively.

### 2.3. Data collection method

The survey was conducted by scanning the two-dimensional code on the network platform *via* the Humanistic Nursing Professional Committee of China Life Care Association. After obtaining the consent of the nursing department and secretary of each hospital, the investigation team first gave the questionnaire to the head nurse of the investigation department of different hospitals and departments across the country and explained the purpose of the questionnaire. The head nurse of the department is responsible for organizing the patients in the department to ensure that they scan the two-dimensional code and fill in the questionnaire. Each patient could participate only once. All items could only be submitted after completion, and the questionnaire was filled out anonymously and independently. Data entry was done by two researchers alone, who checked the original questionnaire data after input to guarantee the accuracy of the data. Missing data or poor-quality questionnaires were eliminated. There were a total of 31,095 questionnaires in this survey, of which 29,108 valid questionnaires were recalled, with an effective completion rate of 93.6%.

### 2.4. Statistical methods

The questionnaire results were input into Excel to create the original database. After removing invalid questionnaires, the data were imported into SPSS 25.0 for statistical analysis. The two-person cross-check method was used to reduce errors and ensure the accuracy of the data inputted. Pearson correlation analysis was used to analyse the correlations between the variables. A hierarchical linear regression equation was used to analyse the effect of relational care on satisfaction with nurses' care. *P* < 0.05 was considered statistically significant.

## 3. Results

A total of 29,108 valid questionnaires were collected from 30 provinces in China. There were 1,910 (6.6%) participants in Northeast China, 5,700 (19.6%) in East China, 15,911 (54.7%) in Central China, and 5,587 (19.2%) in West China.

### 3.1. Current situation of patients' satisfaction with nursing care

The average score of nurse–patient relational care was 4.38 ± 0.57, and the average score of patients' satisfaction with nursing care was 5.40 ± 0.86. The average scores of 12 aspects of nursing relationship satisfaction ranged from 5.11 to 5.49. Among them, patients' satisfaction with nursing ability (5.49 ± 0.89) and emotional support (5.47 ± 0.88) were higher, while patients' satisfaction with family participation (5.11 ± 1.28) and spiritual environment (5.35 ± 1.04) were lower.

### 3.2. Comparison of differences in the nursing care satisfaction scores of subjects with different demographic characteristics

There were statistical differences in the nursing care satisfaction of different patients in terms of gender, age, marital status, education level, residence, family monthly income, department of treatment, type of medical insurance and region. However, there were no statistical differences regarding whether the patients underwent an operation. Briefly, male patients, older patients, married patients, patients without tertiary education, urban patients, patients with high income, surgical patients, high Medicare patients, and patients in Northeast China reported higher satisfaction with nursing care. See Table 1 for more details.

**Table 1:** Comparison of nursing care satisfaction scores of subjects with different demographic characteristics.

**Project**	**Grouping**	**Numbers**	**Score (*x* ±*s*)**	**Statistics**	***p***
Gender	Male	12,049	5.43 ± 0.85	4.77^a^	<0.001
	Female	17,059	5.38 ± 0.87		
Age	18–34	8,334	5.34 ± 0.91	44.46^b^	<0.001
	35–39	13,247	5.41 ± 0.84		
	≥60	7,527	5.47 ± 0.82		
Marriage	Unmarried	24,066	5.36 ± 0.90	9.20^b^	<0.001
	Married	4,001	5.41 ± 0.85		
	Other	1,041	5.33 ± 0.94		
Level of education	Primary school the following	4,334	5.42 ± 0.86	3.15^b^	0.013
	Junior high school	7,128	5.42 ± 0.85		
	High school/technical secondary school	5,601	5.40 ± 0.87		
	College	4,765	5.37 ± 0.88		
	Bachelor degree or above	7,280	5.40 ± 0.86		
Location	City	15,769	5.43 ± 0.85	17.67^b^	<0.001
	Towns	5,983	5.39 ± 0.88		
	Rural	7,356	5.36 ± 0.87		
Per capita monthly household income (Yuan)	<3,000	7,958	5.36 ± 0.88	10.65^b^	<0.001
	3,000– <5,000	9,771	5.40 ± 0.86		
	5,000– <8,000	6,132	5.44 ± 0.83		
	>8,000	5,247	5.42 ± 0.86		
Department	Internal medicine	13,989	5.41 ± 0.85	18.03^b^	<0.001
	Surgical	9,252	5.45 ± 0.83		
	The department of obstetrics and gynecology	2,953	5.32 ± 0.92		
	Pediatric	919	5.30 ± 0.93		
	The critical	731	5.25 ± 0.90		
	Outpatient service	605	5.27 ± 0.94		
	Other	659	5.48 ± 0.85		
Health care type	Health care in cities and towns	16,022	5.42 ± 0.85	14.95^b^	<0.001
	The city health care	6,915	5.42 ± 0.84		
	Provincial health care	1,986	5.46 ± 0.80		
	Commercial insurance	255	5.35 ± 0.86		
	At public expense	439	5.41 ± 0.86		
	At his own expense	3,077	5.27 ± 0.95		
	Other	414	5.35 ± 0.90		
Area	Northeast	1,910	5.50 ± 0.83	50.03^b^	<0.001
	East	5,700	5.43 ± 0.85		
	Middle	15,911	5.42 ± 0.84		
	West	5,587	5.28 ± 0.93		
Operation	Yes	17,607	5.40 ± 0.86	−2.36^a^	0.18
	No	11,501	5.42 ± 0.85		

^a^t-value.

^b^F-value.

### 3.3. Correlation analysis of relational care, scores of each dimension, and nursing care satisfaction

The total mean score of relational care and its dimensions were positively correlated with total satisfaction with nursing care (*r* = 0.35–0.37, *p* < 0.01), and the trust dimension showed the lowest coefficient (*r* = 0.35). In contrast, the care dimension and relationship care were tied for the highest coefficient as shown in Table 2.

**Table 2:** Correlation between relational care, scores of each dimension and nursing care satisfaction (*n* = 29,108).

	**Variable**	**1**	**2**	**3**	**4**	**5**
1	Trust	1				
2	Care	0.94	1			
3	The professional ethics	0.93	0.96	1		
4	Relationship of care	0.97	0.99	0.98	1	
5	Satisfaction with nursing care	0.35	0.37	0.36	0.37	1

All P < 0.01.

### 3.4. Hierarchical linear regression analysis of the effect of relational care on nursing care satisfaction

First, gender, age, marital status, education level, place of residence, family monthly income, department of medical treatment, type of medical insurance, and region were used as the control variables, relational care was taken as the independent variable, and care satisfaction was taken as the dependent variable in the hierarchical linear regression analysis. The specific variables are shown in Table 3. The analysis found that age, region, and the caring, trust, and professional ethics dimensions of relational care were the relevant factors predicting nursing care satisfaction (*p* < 0.05). Therefore, to simplify the regression equation, age and region were included as control variables, and other irrelevant variables were removed. The results of the hierarchical linear regression showed that after controlling for age and region, the dimensions of caring, trust, and professional ethics of relational care significantly predicted nursing care satisfaction, as shown in Table 4.

**Table 3:** Value assignment of each variable.

**Variable**	**Assignment way**
Gender	Male = 0; female = 1
Age	18–34 = 1; 35–59 = 2; ≥60 = 3
Marriage	Married = 1; unmarried = 2; other = 3
Level of education	Primary school and below = 1; junior high school = 2; high school/technical secondary school = 3; college = 4; bachelor degree or above = 5
Location	City = 1; towns = 2; rural = 3
Monthly household income	<3,000 = 1; 3,000– <5,000 = 2; 5,000– <8,000 = 3; ≥8,000 = 4
Department	Internal medicine = 1; surgical = 2; obstetrics and gynecology = 3; pediatric = 4; emergency and critical care unit = 5; outpatient service = 6; other = 7
Health care type	Health care in cities and towns = 1; the city health care = 2; provincial health care = 3; commercial insurance = 4; at public expense = 5; own expense = 6; other = 7
Area	Northeast = 1; east = 2; in the middle = 3; in the west = 4
Operation	Yes = 1; no = 0

**Table 4:** Hierarchical linear regression analysis of nursing relationship satisfaction (*n* = 29,108).

**Layered**	**The independent variables**	**Regression coefficient**	**Standard error**	**Standard regression coefficient**	***t***	***p***
The first layer	(Constant)	5.47	0.02	—	234.96	<0.001
	Age	0.06	0.01	0.05	9.25	<0.001
	Location	−0.07	0.01	−0.06	−10.28	<0.001
The second layer	(Constant)	3.04	0.04	—	71.43	<0.001
	Age	0.03	0.01	0.03	5.38	<0.001
	Location	−0.03	0.01	−0.03	−5.80	<0.001
	Care	0.35	0.03	0.24	10.88	<0.001
	Trust	0.13	0.02	0.09	5.45	<0.001
	The professional ethics	0.07	0.03	0.05	2.14	0.032

The first layer, R^2^ = 0.007, F = 97.148, P < 0.001; second layer, R^2^ = 0.139, ΔR^2^ = 0.132, F = 943.143, P < 0.001.

## 4. Discussion

### 4.1. Patients' satisfaction with nursing care is at an intermediate level

The survey results of 29,108 patients showed that the average patients' satisfaction with nursing care score was 5.40 ± 0.86, which was above the medium level, similar to the research results of Jia et al. (19). Several studies have shown that benevolence, altruism, and dedication are the main concepts of religious traditions (20). Liu's research found that inpatients believed that the strongest care support was the humanitarian care provided by nursing staff (21). This study showed that patients had the highest satisfaction with nursing ability and emotional support, while they had low satisfaction scores with family involvement and spiritual environment. This indicates that patients are satisfied with nurses' professional knowledge and skills, and emotional support provided, and feel safe in their care. However, patients hope that their families can participate in the treatment and nursing. Existing studies have pointed out that the humanistic care ability of clinical nurses is at a relatively low level overall (22, 23). As nurses are busy with clinical treatment, nursing managers pay more attention to the improvement of clinical operation skills, and there is a lack of humanistic care education for clinical nurses (24). Therefore, it is necessary to strengthen nursing staff's keen observation skills. Moreover, patients will undergo physical and psychological changes after illness. In addition to receiving medical treatment and nursing operations, they require emotional support and spiritual comfort from nursing staff. It is very easy for nursing staff to assess the psychological state of patients because they have more contact with them. Nursing staff should therefore help patients increase their self-confidence to overcome illness and provide them with professional and emotionally supportive holistic care.

### 4.2. Scores of nursing care satisfaction in relation to different demographic characteristics

Age, gender, marital status, education level, place of residence, region, family monthly income, type of medical insurance, and department were associated with differences in nursing care satisfaction scores. Patients older than or equal to 60 years old had higher scores, which indicates that patients of this age have lower requirements than younger patients, so they are more satisfied with the evolving humanistic care of nurses. The results of this survey showed that the caring ability of nurses improved with age and experience. Milutinović et al. performed a survey on nursing satisfaction among inpatients in Serbia and found that age influenced nursing satisfaction, which is consistent with the findings of this study (25). Previous study has found that clinical nurses over 35 years old have the highest humanistic care ability, and as they shoulder the social responsibility of caring for the elderly and raising children, so they have more experience in caring for their relatives. Therefore, they are more likely to empathize with patients and give them more care. However, nurses under the age of 35 do not have as much social experience and lack nursing experience, communication skills, and professional knowledge, which affects their caring ability and perception to different degrees (26). Therefore, it is necessary to further improve the humanistic caring ability of nurses under the age of 35. A nurse's responsibility is closely related to their professional qualification and ability. Senior nurses are more likely to build good relationships with patients because of their deep knowledge and vast experience. In contrast, junior nurses lack clinical nursing experience because of their shorter job tenure and so have difficulty in establishing good relationships with patients (27). These findings are consistent with the results of Simmons' finding that humanistic care ability increases with work experience (28). This suggests that humanistic nursing care education should focus on nurses with junior seniority and a professional title (27). Therefore, more management and family support are needed for nurses who are younger than 35. Moreover, male patients were significantly more satisfied with their care than females, which may be because women are more sensitive compared to men. Regarding marital status, those who were married had the highest satisfaction scores. Studies in China show (29) that married couples are happier than unmarried couples. Separated and divorced people had lower happiness scores, and those who are widowed had the lowest. In terms of residence, patients from urban areas scored higher than those from rural areas. In terms of income, those with a middle income scored higher. Regarding education, those below junior high school scored higher, and those with a low education level were more likely to be satisfied. In terms of the type of medical insurance, patients with provincial and municipal medical insurance had higher scores for nursing care satisfaction, while patients without insurance had the lowest scores. When patients without insurance have to pay for services, they become more critical or demanding about the service they receive. In terms of departments, other departments had the highest score, followed by surgery. Patients in surgery have faster recovery times and shorter hospital stays. During treatment, patients pay more attention to their own diseases and are easily satisfied with the humanistic nursing care. Studies in different countries and regions have shown that clinical nurses in intensive care units and emergency departments are more likely to demonstrate altruism (27–30) because they have a higher level of humanistic care compared with clinical nurses in other departments and pay more attention to the health needs of different patients. However, due to the critical condition of patients in the acute and intensive care units, there are few opportunities to communicate with nurses, and physical pain is difficult to communicate with them. Therefore, even if the nurses have a strong caring ability and pay attention to humanistic care, some specific departments will occasionally be associated with lower nursing satisfaction score. In contrast to inpatients who are more concerned about the quality of humanistic care services, outpatients are often only concerned about the hardware facilities and so on. In conclusion, we should particularly focus on women, single, divorced, or widowed patients, as well as outpatients and patients in intensive care unit, emergency, gynecological, and pediatric departments. These populations warrant further humanistic care services.

### 4.3. Nurse–patient relationship predicts patients' satisfaction with nurses

The results showed that the nurse–patient care relationship was positively correlated with nursing care satisfaction (*r* = 0.37, *p* < 0.001). The results of the hierarchical linear regression analysis showed that after controlling for the demographic variables, the caring, trust, and professional ethics dimensions of relational care were the factors predicting nursing satisfaction. The interaction between the patient and nursing staff, eye contact, and respect for the patient's choice can cultivate trust between the nurse and patient, as patients prefer the nurse to treat them as a person rather than a disease. This kind of good care relationship will inevitably affect patients' satisfaction with nurses' care. However, because nursing care satisfaction is a part of patient satisfaction and affected by many factors, the relevant factors explained only 37% of the variance. Hu (31) suggested that the humanistic care ability of nurses can be increased by improving the nursing humanistic care education and evaluation system, creating a harmonious working atmosphere, improving work–family support for nurses, optimizing the allocation of human resources, and accelerating the construction of intelligent nursing. A good working atmosphere is conducive to improving the satisfaction of nursing staff and making them happier and more patient when treating patients. Managers should consider the work quality, nursing service, professional assessment, and other conditions of nursing staff in terms of performance pay and implement humanistic management methods. This will enhance collaboration and trust between care leadership and immediate bedside staff to create a culture of care that improves the support, communication, and opportunities for shared decision-making. Establishing a good humanistic care atmosphere will trigger a positive professional identity and improve nurses' enthusiasm for work (32).

## 5. Conclusion

This study investigated the correlation between patients' satisfaction with nursing care and the nurse–patient care relationship in Chinese hospitals through a multicentre survey. Patients' satisfaction with nursing care in Chinese hospitals was positively correlated with the nurse–patient care relationship and these results may provide guidelines for the establishment of relationship care systems. However, patient care satisfaction is related to any group or individual that affects the process and outcome of medical activities. These factors include governments, hospitals, and patients themselves. Nurse–patient relationship care is only one factor influencing patients' nursing care satisfaction. To build a strong nurse–patient relationship and improve nursing care satisfaction, it is necessary to establish a nursing care educational system in hospitals and so increase humanistic care knowledge training. Since 2019, Liu has led experts in the field of humanistic nursing to formulate the expert consensus on “Norms of Practice of Humanistic Care in Hospital Nursing” and the group standard of “Norms of Humanistic Care Management in Ward Nursing” (33–36). Moreover, the Chinese Journal of Hospital Management and the National Standard Network formally published and selected 107 hospitals around China to start the application of “consensus” and “standards.” They also created training criteria for the humanistic nursing care in 107 hospitals, and provided a basis and reference for the next step of a cross-sectional survey for humanistic care intervention. However, this study has several limitations. For example, this study only focused on some departments in the hospital. Future studies will need to include other departments, such as the orthopedics, cardiovascular, and tumor departments.

## Data availability statement

The raw data supporting the conclusions of this article will be made available by the authors, without undue reservation.

## Ethics statement

Ethical review and approval was not required for the study on human participants in accordance with the local legislation and institutional requirements. Written informed consent to participate in this study was provided by the participants' legal guardian/next of kin. Ethical approval for this study was obtained through our university's Research Ethics Board (2022-S161).

## Author contributions

SG, YC, XH, and YL: study conception and design. SG, YC, and HC: acquisition of data. QZ, XH, and BS: analysis and interpretation of data. YL and BS: critical revision. All authors contributed to the article and approved the submitted version.
